# Exploring the role of metabolic pathways in TNBC immunotherapy: insights from single-cell and spatial transcriptomics

**DOI:** 10.3389/fendo.2024.1528248

**Published:** 2025-01-09

**Authors:** Shi-liang Chen, Yi-Ran Fei, Xin-xian Cai, Cong Wang, Shi-yuan Tong, Zhe-zhong Zhang, Yan-xia Huang, Dan-dan Bian, Yi-bo He, Xiao-xiao Yang

**Affiliations:** ^1^ The First Affiliated Hospital of Zhejiang Chinese Medical University (Zhejiang Provincial Hospital of Chinese Medicine), Hangzhou, China; ^2^ The First Clinical Medical College, Zhejiang Chinese Medical University, Hangzhou, China; ^3^ School of Medical Technology and Informmation Engineering, Zhejiang Chinese Medical University, Hangzhou, China; ^4^ State Key Laboratory of Medical Neurobiology and MOE Frontiers Center for Brain Science, Institutes of Brain Science, Fudan University, Shanghai, China

**Keywords:** triple-negative breast cancer, metabolic reprogramming, immune cell function, immune checkpoint inhibitors, combination therapies, tumor microenvironment

## Abstract

The article provides an overview of the current understanding of the interplay between metabolic pathways and immune function in the context of triple-negative breast cancer (TNBC). It highlights recent advancements in single-cell and spatial transcriptomics technologies, which have revolutionized the analysis of tumor heterogeneity and the immune microenvironment in TNBC. The review emphasizes the crucial role of metabolic reprogramming in modulating immune cell function, discussing how specific metabolic pathways, such as glycolysis, lipid metabolism, and amino acid metabolism, can directly impact the activity and phenotypes of various immune cell populations within the TNBC tumor microenvironment. Furthermore, the article explores the implications of these metabolic-immune interactions for the efficacy of immune checkpoint inhibitor (ICI) therapies in TNBC, suggesting that strategies targeting metabolic pathways may enhance the responsiveness to ICI treatments. Finally, the review outlines future directions and the potential for combination therapies that integrate metabolic modulation with immunotherapeutic approaches, offering promising avenues for improving clinical outcomes for TNBC patients.

## Introduction

Triple-negative breast cancer (TNBC) treatment has historically relied on chemotherapy due to the absence of targeted therapies, limiting effective options. Recent advancements in immunotherapy, particularly immune checkpoint inhibitors (ICIs) like PD-L1 inhibitors, have shown potential, as evidenced by trials such as IMpassion130 (1, 2). However, the modest efficacy of ICIs, benefiting only a subset of patients, highlights the challenges TNBC’s heterogeneity poses. Identifying predictive biomarkers and exploring combination strategies, including metabolic interventions, are critical to improving therapeutic outcomes and addressing TNBC’s metabolic pathways (3).

Although TNBC is generally considered a “cold” tumor with limited immune cell infiltration, emerging evidence suggests it has antigenic properties conducive to immunotherapy (4). TNBC generally shows low levels of tumor-infiltrating lymphocytes (TILs); the presence of specific immune cell types can correlate with better patient outcomes. Specific immune markers, such as granzyme B^+^ CD8^+^ T cells (5), sometimes correlate with improved prognosis. Additionally, plasma cells and other immune subsets have been linked to survival benefits (6), challenging the traditional view of TNBC as uniformly immunologically inactive. Understanding this heterogeneity is crucial for tailoring immunotherapies to re-engage the immune system effectively (7).

ICIs, which block proteins like PD-1 that suppress immune responses, have emerged as promising therapies for TNBC. Cytotoxic T lymphocytes (CTLs) play a pivotal role in anti-tumor immunity, while regulatory T cells (Tregs) can hinder these responses. PD-1^+^ CTLs and other tumor-infiltrating lymphocytes (TILs) significantly impact ICI efficacy. A nuanced understanding of the interactions between immune cell populations within TNBC is vital for optimizing immunotherapeutic strategies. Advancements in single-cell RNA sequencing (scRNA-seq) and spatial transcriptomics have revolutionized TNBC research (8). These technologies provide unprecedented insights into tumor heterogeneity and immune microenvironments by analyzing gene expression at single-cell resolution and mapping spatial interactions (9–11). Studies reveal diverse immune cell subsets and spatial relationships, offering new biomarkers and therapeutic targets (12). Integrating these technologies enables researchers to uncover immune evasion mechanisms and develop tailored immunotherapeutic strategies.

Applying these advanced techniques has deepened the understanding of TNBC’s tumor microenvironment and immune interactions. Researchers can better predict therapeutic responses and personalize treatment (13) by identifying cellular diversity and spatial organization. These insights hold significant potential for identifying novel targets, improving clinical outcomes, and advancing precision medicine in TNBC.

## Metabolic influences on immune cells in the tumor microenvironment

### Link between metabolic pathways and immune function

The metabolic landscape within the tumor microenvironment (TME) significantly impacts the behavior and functionality of immune cells. Tumors often exhibit altered metabolic pathways, producing specific metabolites that can modulate immune responses (14, 15). For instance, it has been observed that the accumulation of lactate, a byproduct of glycolysis, can create an immunosuppressive environment by inhibiting the function of cytotoxic T cells and promoting regulatory T cells (16). Furthermore, tumor-derived metabolites such as adenosine can disrupt T cell activation and promote immune evasion mechanisms (17). This dynamic interplay creates a feedback loop in which tumor cells’ metabolic state affects their proliferation and survival and influences the immune landscape, leading to an environment conducive to tumor progression (18).

Recent studies have elucidated how specific metabolic pathways in tumor cells can directly alter the immune response. For example, it was found that activating IDO pathway in tumors results in tryptophan catabolism, leading to T cell dysfunction and promoting an immune-suppressive environment (19). Moreover, the Warburg effect, characterized by increased aerobic glycolysis in tumor cells, has created an environment that favors the recruitment of immunosuppressive cell types while inhibiting effector T cell functions (20). Understanding these metabolic interactions is crucial for developing strategies to reprogram the TME to reinvigorate anti-tumor immunity (21).

### Importance of metabolic pathways in modulating immune response

Metabolic reprogramming is emerging as a critical factor influencing immune cell functionality and their therapeutic responses. Immune cells adapt their metabolism to fulfill their bioenergetic and biosynthetic needs during activation. For instance, T cells require metabolic reprogramming towards glycolysis to sustain their proliferation and effector functions. However, a skewed metabolic environment can lead to dysfunction (16). Furthermore, studies have shown that targeting metabolic pathways enhances the immune response against TNBC. By inhibiting metabolic checkpoints like mTOR and AMPK, it is possible to improve T cell activation and restore anti-tumor immunity (5).

In the context of TNBC, therapeutic strategies focusing on metabolic reprogramming show promise in enhancing the efficacy of existing treatments. Combining metabolic inhibitors with immunotherapy has been proposed as a novel approach to improve the anti-tumor immune response. For instance, recent research highlights the potential of using metabolic modulators to enhance the effectiveness of immune checkpoint inhibitors, which could lead to better clinical outcomes for TNBC patients (19). Overall, understanding the intricate relationship between metabolic pathways and immune function presents an opportunity to develop innovative strategies to augment the effectiveness of therapies to TNBC.

## Metabolic pathways and their effects on immune cells in TNBC

### Overview of key metabolic pathways

**Figure 1:**
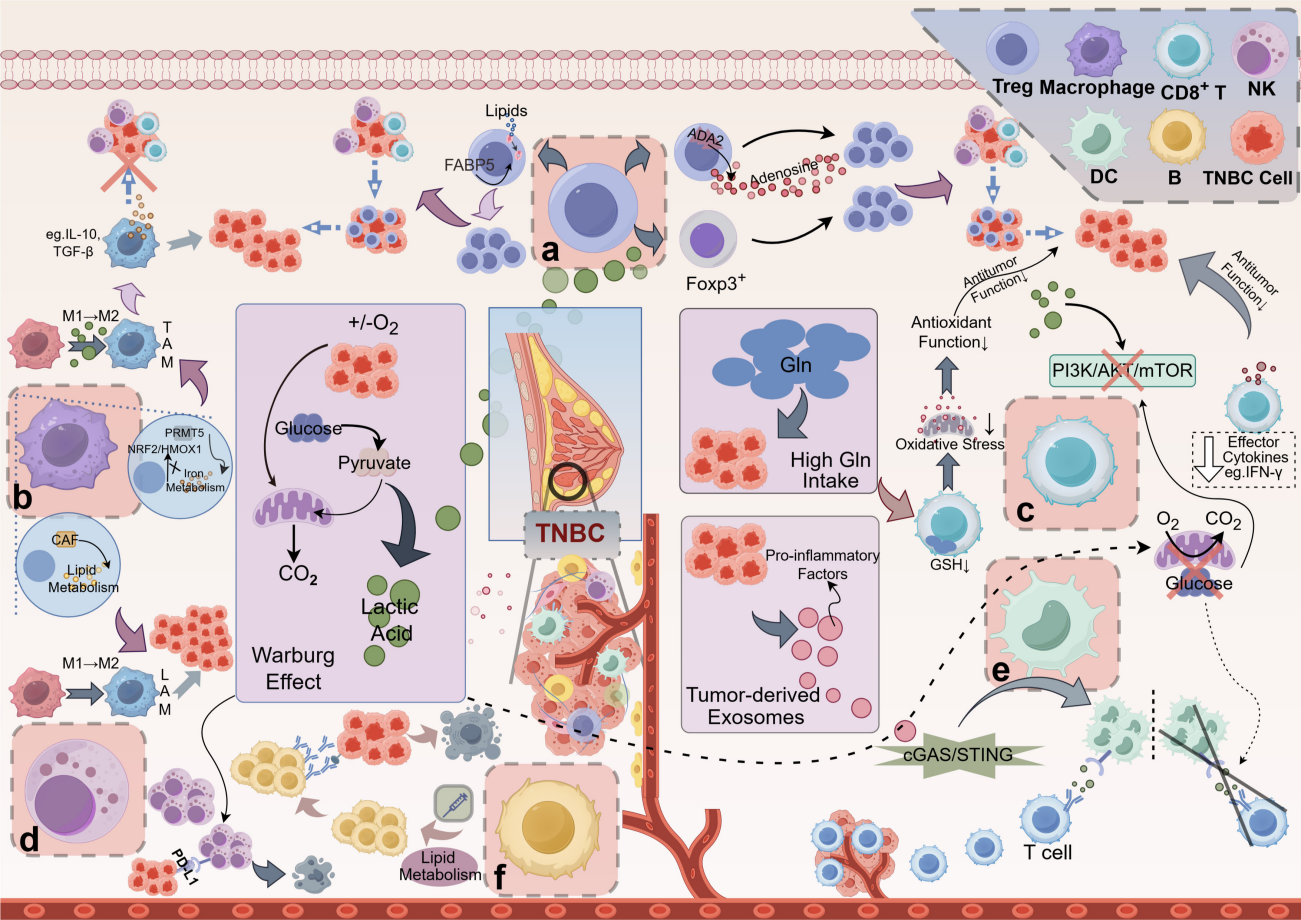
Mechanisms of Metabolite-Mediated Immunosuppression in the TNBC. **(A)** Tregs: Lactic acid boosts FOXP3 expression in Tregs, enhancing their proliferation and dominance in the TME, which inhibits CD8^+^ T cells and NK cells, promoting tumor immune evasion. Adenosine, produced via ADA2 on Tregs, further reinforces Treg predominance, contributing to the immunosuppressive TME. Lipid metabolites, transported by proteins like FABP5, also support Treg proliferation and function. **(B)** Macrophages: Lactic acid drives macrophage polarization to the immunosuppressive M2 phenotype, forming TAMs that secrete IL-10 and TGF-β, suppressing antitumor responses. PRMT5 regulates iron metabolism, inhibiting pro-inflammatory M1 macrophages and favoring M2 polarization. CAF-induced lipid metabolism upregulation further promotes M2 macrophage transformation. (C) CD8^+^ T cells: High lactic acid in the TNBC microenvironment lowers local pH and disrupts the PI3K/AKT/mTOR pathway, impairing CD8^+^ T cell proliferation and cytokine secretion (e.g., IFN-γ), weakening antitumor immunity. Glucose consumption via the Warburg effect depletes glucose needed for glycolysis in CD8^+^ T cells, exacerbating this inhibition. Additionally, high Gln uptake by TNBC cells reduces GSH synthesis in CD8^+^ T cells, impairing oxidative stress tolerance and antitumor function. **(D)** NK cells: Lactic acid increases PD-L1 expression on tumor cells, binding PD-1 on NK cells and inhibiting their cytotoxicity, leading to NK cell exhaustion and tumor immune escape. **(E)** DCs: TNBC cells release exosomes that activate dendritic cells via the cGAS/STING pathway, enhancing T cell activation and immune responses. **(F)** B cells: Lipid metabolism significantly impacts B cell function, particularly in the context of antibody production and memory formation. However, metabolic reprogramming, such as the Warburg effect, reduces glycolysis in DCs, impairing their maturation and antigen presentation, thus weakening T cell activation and effector immune cell infiltration in the tumor. TNBC, Triple-Negative Breast Cancer; Treg, Regulatory T Cell; FOXP3, Forkhead Box P3; TME, Tumor Microenvironment; NK cell, Natural Killer Cell; ADA2, Adenosine Deaminase 2; FABP5, Fatty Acid-Binding Protein 5; TAM, Tumor-Associated Macrophage; IL-10, Interleukin 10; TGF-β, Transforming Growth Factor Beta; PRMT5, Protein Arginine Methyltransferase 5; CAF, Cancer-Associated Fibroblast; LAM, Lipid-Associated Macrophage; PI3K, Phosphoinositide 3-Kinase; AKT, Protein Kinase B (often referred to as AKT); mTOR, Mechanistic Target of Rapamycin; IFN-γ, Interferon Gamma; Gln, glutamine; GSH, Glutathione; PD-L1, Programmed Death-Ligand 1; PD-1, Programmed Death-1; DC, Dendritic Cell; cGAS, Cyclic GMP-AMP Synthase; STING, Stimulator of Interferon Genes. This figure was created using the Figdraw online drawing tool.

Metabolic pathways are critical determinants of immune cell function and can significantly influence the efficacy of anti-tumor responses in TNBC. Key metabolic processes, including glycolysis, lipid metabolism, and amino acid metabolism, orchestrate the activities of various immune cells (**Figure 1**). Glycolysis, for instance, is vital for T cell activation and proliferation. Increased glycolytic activity in T cells correlates with enhanced effector functions, allowing them to respond effectively to tumor cells (22). In contrast, fatty acid oxidation is crucial for the developing and maintaining memory T cells, ensuring long-lasting immune protection against recurrent tumors (23).

Amino acid metabolism also plays a pivotal role in immune responses. The availability of specific amino acids, such as glutamine, influences T cell metabolism and function. Tumor cells often deplete local amino acids, leading to T cell dysfunction and impaired anti-tumor activity (24). Understanding these metabolic pathways provides insights into how metabolic reprogramming in immune cells can enhance their functionality and effectiveness against TNBC.

### Effects on different immune cells

#### Regulatory T cells

Lactate accumulation enhances the immunosuppressive function of Treg cells by activating FOXP3 gene expression (25). Lactate also induces Treg cell proliferation, allowing them to dominate within the tumor microenvironment, further diminishing the activity of CD8^+^ T cells, NK cells, and thereby supporting immune evasion by the tumor. Adenosine, catalyzed by ADA2 (Adenosine Deaminase 2), activates the A2A receptor on Treg cells, enhancing their immunosuppressive functions (26). Elevated adenosine levels strengthen Treg cell function and reduce effector T cell activation, fostering an immunosuppressive environment. Treg cell metabolism relies on specific lipid metabolic pathways, with these metabolites promoting Treg immunosuppressive abilities through particular lipid transport proteins, such as FABP5 (27). Lipid accumulation in Treg cells facilitates their proliferation and survival, further diminishing the activity of effector T cells within the tumor microenvironment (28).

#### Macrophages

The metabolic profiles of macrophages are critical in dictating their pro-tumor or anti-tumor functions. In TNBC, metabolic reprogramming within macrophages can lead to polarization towards a tumor-promoting M2 phenotype characterized by immunosuppressive properties (29). Conversely, promoting metabolic shifts towards an M1-like state can enhance their anti-tumor capabilities. Understanding these metabolic dynamics could lead to novel strategies for reprogramming macrophages to adopt anti-tumor phenotypes, potentially improving therapeutic outcomes in TNBC. Lactate induces macrophage polarization towards an immunosuppressive M2 phenotype, giving rise to tumor-associated macrophages (TAMs) (30, 31). These M2-polarized macrophages secrete elevated levels of immunosuppressive factors, such as IL-10 and TGF-β, which inhibit the antitumor responses of T cells and NK cells (32). PRMT5, by modulating iron metabolism, restricts the pro-inflammatory activity of M1 macrophages, thereby allowing the immunosuppressive properties of the M2 phenotype to predominate (33). Reducing in iron ions further promotes M2 polarization by inhibiting the NRF2/HMOX1 pathway. CAFs upregulate lipid metabolism, driving macrophages toward a lipid-associated macrophage (LAM) phenotype (34), forming immunosuppressive macrophages. These macrophages enhance immunosuppressive effects through lipid signaling molecules, reducing the functional infiltration of effector immune cells within the tumor.

#### CD8^+^ T cells

Within the tumor microenvironment, the accumulation of high concentrations of lactate results in functional impairment of CD8^+^ T cells by lowering the local pH. TNFR2 enhances immunosuppressive capacity in endothelial cells by inhibiting the glycolytic pathway, resulting in decreased CD8^+^ T cell activity. Blocking TNFR2, however, can restore antitumor immunity (30, 35). Lactate interferes with the mTOR signaling pathway, inhibiting T cell proliferation and diminishing the secretion of key effector cytokines, such as IFN-γ, further compromising antitumor immunity (31). Through the Warburg effect, TNBC cells preferentially consume glucose, leading to glucose deprivation in the surrounding environment, which hampers CD8^+^ T cells’ ability to maintain the glucose levels required for efficient glycolysis. This glucose deficiency directly reduces the activity of CD8^+^ T cells and, by limiting energy supply through the PI3K/AKT/mTOR pathway (36), decreases their proliferation and cytotoxicity. In the TNBC microenvironment, high glutamine uptake exhausts the glutamine needed by immune cells, adversely affecting particularly the antioxidant-dependent CD8^+^ T cells (37). Glutamine scarcity restricts glutathione synthesis in T cells, reducing their tolerance to oxidative stress and weakening their antioxidative and antitumor functions within the tumor.

#### Natural killer cells

Lactate significantly diminishes the cytotoxicity of NK cells, reducing their tumor-killing capacity. The accumulation of lactate also compromises NK cell survival and proliferation by acidifying the environment, further weakening their immune clearance functions (38). In lung cancer, lactate has been found to upregulate PD-L1 expression on tumor cell surfaces via the Warburg effect (39), suggesting a close link between metabolic reprogramming of tumor cells and immune evasion mechanisms. This pathway promotes NK cell exhaustion, enhancing the tumor’s ability to evade immune detection (40, 41).

#### Dendritic cells

Dendritic cells are pivotal in antigen presentation and the initiation of T cell responses. Tumor-derived metabolites can profoundly influence DC function, affecting their ability to activate T cells effectively. Exosomes secreted by TNBC cells, rich in pro-inflammatory molecules, activate the cGAS/STING pathway in dendritic cells (42, 43), thereby enhancing the initiation of antitumor immune responses. The release of these exosomes bolsters DC activity, facilitating downstream T cell activation and strengthening immune responses within the tumor microenvironment. However, metabolic reprogramming in TNBC suppresses the glycolytic pathway in dendritic cells (44), impairing their maturation and activation capabilities and consequently weakening their efficacy in T cell activation. This metabolic inhibition directly impacts the antigen-presenting capacity of DCs, resulting in reduced infiltration and activity of T cells within the tumor. Researchers can explore therapeutic strategies to enhance DC-mediated T cell activation by understanding how tumor metabolism affects DC function. Targeting metabolic pathways in DCs may help restore their function and improve the overall anti-tumor immune response (45, 46).

#### B cells

Lipid metabolism significantly impacts B cell function, particularly in antibody production and memory formation. Research indicates that B cells rely on fatty acid metabolism for optimal antibody responses (47). In TNBC, modulating lipid metabolic pathways could enhance the effectiveness of therapeutic vaccines by promoting robust B cell activation and differentiation. For example, interventions that enhance lipid uptake and utilization by B cells might increase their ability to produce high-affinity antibodies against tumor antigens (48). This approach could represent a novel strategy to improve vaccine efficacy in TNBC patients.

### Future directions and clinical implications

The interaction between metabolic pathways and immune responses offers promising avenues for combination therapies in TNBC. Studies suggest that metabolic reprogramming can significantly enhance immune cell efficacy against tumors. Combining ICIs with metabolic pathway-targeting agents could boost anti-tumor immunity by reinvigorating T cells or enhancing TIL populations. These strategies may overcome the limitations of ICIs alone and lead to more personalized treatments based on individual metabolic profiles. Advanced techniques like single-cell functional enzymatic assays (scFEA) and metabolic profiling tools (e.g., Mebocost, scMetabolism) enable more insights into immune cell metabolism in TNBC. These tools allow single-cell analysis of metabolic activity, helping researchers understand how immune cells metabolize nutrients within the tumor environment. By mapping the metabolic landscape, researchers can identify critical metabolic checkpoints as therapeutic targets, which could lead to optimized immune function therapies. The goal of immune metabolism research in TNBC is to translate findings into clinical practice. Targeting specific metabolic pathways, such as glycolysis, could lead to personalized therapies that improve survival and quality of life for TNBC patients. Collaboration between researchers and clinicians is essential, with clinical trials for combination therapies already underway, signaling a shift towards personalized medicine in TNBC and improved treatment outcomes.

## Conclusion

Metabolic products, such as lactate and adenosine, are pivotal in establishing an immunosuppressive tumor microenvironment by modulating immune cell functions. Lactate has been shown to promote the proliferation of Tregs while impairing the functionality of cytotoxic CD8^+^ T cells, and adenosine disrupts T cell activation via the A2A receptor. Recent research further highlights the impact of glutamine depletion on T cell oxidative stress tolerance, demonstrating the intricate connection between metabolic reprogramming and immune responses in TNBC. These findings underscore the potential of combining ICIs with metabolic modulators targeting pathways such as glycolysis and fatty acid oxidation. Preclinical models indicate that such combination therapies can effectively reinvigorate exhausted T cells and enhance antitumor immunity, paving the way for improved therapeutic strategies.
